# Retinal Morphology and Sensitivity Are Primarily Impaired in Eyes with Neuromyelitis Optica Spectrum Disorder (NMOSD)

**DOI:** 10.1371/journal.pone.0167473

**Published:** 2016-12-09

**Authors:** Ryutaro Akiba, Hirotaka Yokouchi, Masahiro Mori, Toshiyuki Oshitari, Takayuki Baba, Setsu Sawai, Satoshi Kuwabara, Shuichi Yamamoto

**Affiliations:** 1 Department of Ophthalmology and Visual Science, Chiba University Graduate School of Medicine, Chiba, Chiba, Japan; 2 Department of Neurology, Chiba University Graduate School of Medicine, Chiba, Chiba, Japan; University of Miami, UNITED STATES

## Abstract

**Background:**

Previous studies of neuromyelitis optica spectrum disorder (NMOSD) using spectral domain optical coherence tomography (SD-OCT) showed that the outer nuclear layer (ONL) in eyes without a history of optic neuritis (ON) was thinner than that of healthy controls. It remains unclear whether the ONL thinning is caused by a direct attack on the retina by an autoantibody or a retrograde degeneration.

**Objective:**

To determine the mechanisms involved in the retinal damage in eyes with NMOSD without ON.

**Methods:**

SD-OCT was used to determine the thicknesses of the different retinal layers of 21 eyes of 12 NMOSD patients without prior ON and 19 eyes of 10 healthy controls. Eyes with peripapillary retinal nerve fiber layer (RNFL) thinning were excluded to eliminate the confounding effects of retrograde degeneration. Microperimetry was used to determine the central retinal sensitivity. The data of the two groups were compared using generalized estimated equation models to account for inter-eye dependencies.

**Results:**

The ganglion cell plus inner plexiform layer and the inner nuclear layer plus outer plexiform layer thicknesses of the NMOSD eyes were not significantly different from that of the control eyes (*P* = 0.28, *P* = 0.78). However, the ONL and average macular thickness (AMT) in the NMOSD eyes were significantly thinner than that of the control eyes (*P* = 0.022, *P* = 0.036). The retinal sensitivity in the central 10°, 10° to 2°, and 2° sectors were significantly lower in the NMOSD eyes than in the control eyes (*P* = 0.013, *P* = 0.022, *P* = 0.002).

**Conclusions:**

The ONL thinning, AMT thinning, and reduced retinal sensitivity in eyes with NMOSD without significant peripapillary RNFL thinning are most likely due to direct retinal pathology.

## Introduction

Neuromyelitis optica (NMO) is an inflammatory autoimmune disease that is associated with severe optic neuritis (ON) and/or longitudinally extensive transverse myelitis (LETM).[1] The discovery of immunoglobulin G (NMO-IgG)[2]/anti-aquaporin 4 (AQP4)[3] antibody has broadened the spectrum of NMO to neuromyelitis optica spectrum disorders (NMOSD).[4]

Optical coherence tomography (OCT) has been used to evaluate the retinal structures in neurogenerative diseases including multiple sclerosis (MS)[5] and NMOSD[6]. Previous studies have reported that the thickness of the peripapillary retinal nerve fiber layer (RNFL) was reduced in MS patients with history of optic neuritis (ON)[5]. It has also been reported that the peripapillary RNFL thickness is reduced in MS patients even with no history of ON[7, 8]. The subclinical changes of the retinal morphology are presented as either unrecognized subclinical optic neuritis or primary retinal pathology.

In NMOSD, Merle et al reported a thinning of the peripapillary RNFL in NMO patients with ON episodes [9] which has been confirmed by two other studies. [6, 10] Interestingly, Merle et al also reported a thinning of the peripapillary RNFL in NMO patients without prior ON history, and the changes were suggested to be due to chronic axonal injuries.[9] Outteryck et al also reported a thinning of the peripapillary RNLF thinning in NMOSD patients without a history of ON, and they hypothesized a retrograde transsynaptic degeneration as the cause of the pathology for subclinical RNFL thinning.[11] Additionally, a thinning of the macular RNFL[12] and ganglion cell and inner nuclear cell layer (GCIP)[13] in patients with NMOSD without a history of ON has been reported. These reports are consistent with previous studies suggesting a subclinical involvement of the anterior visual pathway possibly causing retrograde degeneration. On the other hand, several studies did not find a peripapillary RNFL thinning[10, 14–16], and thus the results of the different studies are contradictory.

Other OCT studies reported a significant thinning of the outer nuclear layer (ONL) in NMOSD patients without prior history of ON.[13, 17] Two causes for the ONL thinning have been proposed; one is that the thinning is due to a primary anti-AQP4-mediated retinal pathology directed against retinal astrocytes,[17] and the second cause is that it is due to retrograde degeneration.[13] These two hypotheses are contradictory, and there has not been sufficient evidence to support either hypothesis.

Parks et al reported that the ONL is thicker in patients with NMOSD than in healthy controls, and Schneider et al reported that the outer retinal layer including the ONL was not significantly different from that of healthy controls. [15, 18] These contradictory findings indicated that the morphological characteristics of NMOSD patient without prior optic neuritis have not been definitively determined.

To evaluate whether there is a primary retinal pathology independent of ON in NMOSD patients, we focused on patients without a prior history of ON. We also excluded eyes with peripapillary RNFL thinning to eliminate the confounding effects of retrograde degeneration. Earlier studies have reported subclinical changes of visual functions.[13, 19] We used macular integrity assessment (MAIA) to evaluate the visual function. We evaluated the morphological and retinal sensitivity changes in eyes with NMOSD without prior optic neuritis and peripapillary RNFL thinning.

## Materials and Methods

### Standard protocol approval, registration, and patient consent

The study was approved by Ethics Committee of Chiba University Hospital, and a written informed consent was obtained from all patients. The procedures used conformed to the tenets of the Declaration of Helsinki.

### Participants

Twenty-one eyes of 12 patients who were diagnosed with NMOSD in the Department of Ophthalmology or Neurology of the Chiba University Hospital were studied (Table 1). A diagnosis of NMOSD was made by the 2015 International Consensus Diagnostic Criteria.[20] Nine of the 12 patients were seropositive for the anti-AQP4 antibody as determined by ELISA. Three anti-AQP4 seronegative patients were confirmed to be seronegative by cell-based assays, and the diagnosis of these patients was based on the presence of acute brainstem syndrome and the area postrema syndrome. The oligo clonal band (OCB) was majored in 10 of the 12 patients, and the samples were tested by isoelectric focusing and silver staining. None of the eyes had a history of optic neuritis.

**Table 1:** Clinical characteristics of patients with NMOSD.

No of Patients	Age	Sex	Laterality	Refractive errors	Phenotype	Anti-AQP4 Antibody	OCB	Disease duration (Month)
**1**	66	F	R	-1	LETM	+	-	103
	66	F	L	-4.75	LETM	+	-	103
**2**	49	F	R	0.25	EN	+	-	16
	49	F	L	0	EN	+	-	16
**3**	61	F	R	-4.75	EN, LETM	+	-	101
	61	F	L	-4.5	EN, LETM	+	-	101
**4**	41	M	R	-2	LETM, ABS, APS	-	-	10
	41	M	L	0.75	LETM, ABS, APS	-	-	10
**5**	43	F	R	-4.25	LETM	+	ND	26
	43	F	L	-3.75	LETM	+	ND	26
**6**	47	F	R	-0.25	LETM	+	-	87
	47	F	L	0	LETM	+	-	87
**7**	63	F	L	2.75	ON in Fellow eye	+	-	44
**8**	38	F	L	-1	ON in Fellow eye	+	ND	91
**9**	49	F	R	-2.5	LETM	+	-	1
	49	F	L	-2.5	LETM	+	-	1
**10**	55	F	L	-1	LETM, ABS, APS, ON in fellow eye	-	-	299
**11**	41	M	R	-0.25	LETM, ABS	-	-	1
	41	M	L	-0.5	LETM, ABS	-	-	1
**12**	45	F	R	0	LETM	+	-	35
	45	F	L	-0.25	LETM	+	-	35
**Average**	49.5			-1.4				56.9
**SD**	8.9			2.1				66.5

Abbreviations: ABS = acute brainstem syndrome; APS = area postrema syndrome; Anti-AQP4 Ab = anti-aquaporin-4 antibody; EN = encephalitis; LETM = longitudinally extensive transverse myelitis; F = Female; M = Male;NMOSD = neuromyelitis optica spectrum disorder; ND = not done; OCB = oligoclonal band; ON = optic neuritis; SD = standard deviation.

Nineteen eyes of 10 volunteers recruited from the Chiba University staff were studied as healthy controls. The history and medical records of all the patients were reviewed to determine co-morbidities and history of optic neuritis.

The exclusion criteria were; history of optic neuritis as determined by examination of the medical records, presence of peripapillary RNFL thinning determined by OCT, myopia higher than -6 diopters, and the presence of glaucoma, cataract, and other retinal diseases. Slit-lamp examinations were performed to exclude obvious ophthalmological disease in our patients.

### Ophthalmological examinations

The best-corrected visual acuity (BCVA) was monocularly measured in all participants using a retro-illuminated Landolt C chart, and the decimal BCVA was converted to the logarithm of the minimal angle of resolution (logMAR) units. The refractive error (spherical equivalent) was measured by a well-trained examiner.

### Optical coherence tomography (OCT)

**Fig 1:**
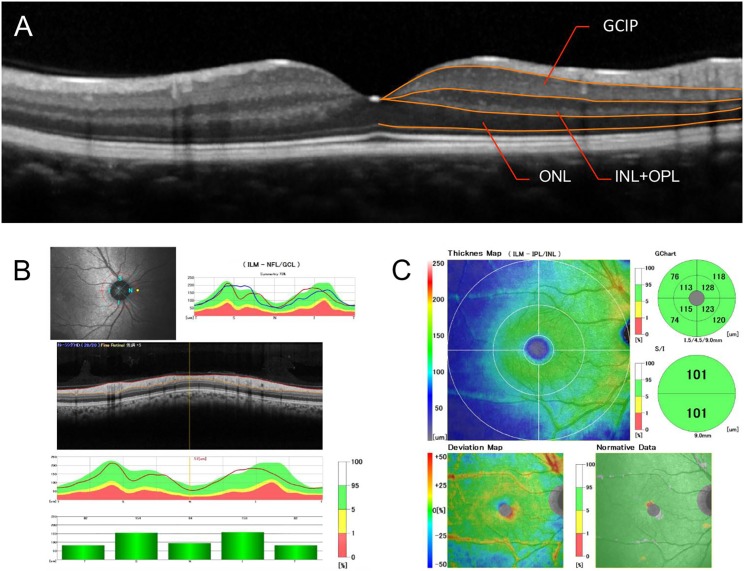
Representative optical coherence tomographic (OCT) images and retinal segmentation of the images. (A) Orange segmentation lines define the ganglion cell layer and inner plexiform layer (GCIP), the inner nuclear layer and outer plexiform layer (INL+OPL), and the outer nuclear layer (ONL). (B) Disc circle mode of OCT was used to evaluate the peripapillary retinal nerve fiber layer thickness around the optic disc. (C) Macula map mode was used to evaluate the thickness of the different retinal layers including the GCIP, INL+OPL, and ONL within a 9 mm x 9 mm area centered on the fovea.

OCT was performed with the RS-3000 Advance (Nidek Corp, Tokyo, Japan) with software version 1.5.0. The RS-3000 is a spectral domain optical coherence tomographic instrument equipped with a confocal scanning laser ophthalmoscope. The RS-3000 scans consisted of 53 000 A-scans/sec and a 4 μm axial resolution which results in a clear differentiation of the different retinal layers (Fig 1A). The optic disc circle mode was used for circular scans around the optic disc to evaluate the peripapillary RNFL thickness (Fig 1B). The macula map mode was used for raster scanning over a 9 mm x 9 mm area centered on the fovea with a scan density of 512 horizontal A-scans. The data obtained from 9 mm radius of the foveal center was used to evaluate the thicknesses of the ganglion cell and inner plexiform layer (GCIP), the inner nuclear layer and outer plexiform layer (INL+OPL), and the ONL (Fig 1C). In addition, the average macular thickness (AMT) within a 3 mm radius of the foveal center was evaluated. The OCT images were recorded without pupillary dilatation by a well-trained examiner. The scans were assessed for the signal strength index (SSI), and images with SSI greater than 50 were analyzed. Scans with non-centered images or poorly colored thickness maps were excluded.

### Exclusion of eyes with peripapillary RNFL thinning

Eyes with peripapillary RNFL thinning were excluded to prevent the confounding effects of retrograde degeneration as has been described.[21] The OCT software used a computerized algorithm to compare the average peripapillary RNFL thickness of each participant with an embedded normative database of age-matched control subjects (ages ≥18 years). This algorithm assigned the measurements as a rank against a normal distribution percentile scheme derived from the database of age-matched controls. The measurements were separated into; normal (5–95th percentile), below normal (<5th percentile), markedly below normal (<1st percentile), or supra-normal (>95th percentile). The internal Nidek RS-3000 normative database consisted of 130 Asian subjects with a mean age of 47.6 ± 15.0 years.[22] Eyes that belonged to groups other than the normal were excluded.

### Optical coherence tomographic segmentation

Automatic measurements of the thicknesses of the intraretinal layers including the peripapillary RNFL, GCIP, and ONL were made, and significance maps were determined by an in-built software of the RS3000. The RNFL thickness was measured between the internal limiting membrane and the outer boundary of the retinal nerve fiber layer. The GCIP thickness was measured between inner boundary of ganglion cell layer and the outer boundary of the inner plexiform layer (IPL). The INL+OPL thickness was measured between inner boundary of inner nuclear layer and the outer boundary of the outer plexiform layer. The ONL thickness was measured between the inner and outer boundary of the outer nuclear layer. Another measure obtained from optical coherence tomography scans centered on the foveal center was the average macular thickness (AMT). The AMT represents the thickness of the retina between the internal limiting membrane and the junction between the inner segments and outer segments of the photoreceptors within a 3 mm radius of the fovea.

### Retinal sensitivity

**Fig 2:**
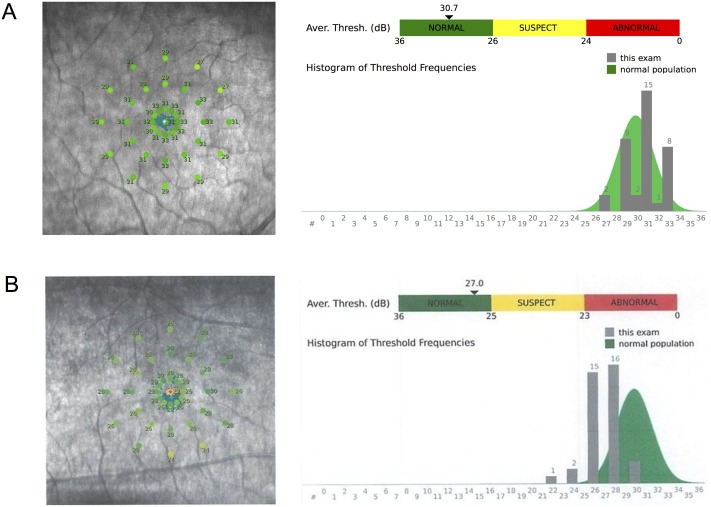
Evaluation of retinal sensitivity. The retinal sensitivities were obtained from 37 points within the central 10 degrees of the foveal center. The average retinal sensitivity of the central 10°, central 10° to 2°, and central 2° were calculated. The macular integrity assessment (MAIA) was used to determine the retinal sensitivity. (A) Retinal sensitivity of a healthy control eye. Retinal sensitivities obtained from each point are shown on the left. The average sensitivity of central 10° and the histogram of threshold frequencies compared with built-in database of normal population are shown on the right. (B) Retinal sensitivity of an eye with neuromyelitis optica spectrum disorder (NMOSD). The patient was a 66-year-old woman who had history of longitudinally extensive transverse myelitis and was positive for serum autoantibody against aquaporin-4. The average retinal sensitivity of the central 10° was 27.0 dB whereas the average sensitivity of healthy control eyes was 29.39 dB.

Earlier studies have reported subclinical changes of the visual functions, e.g., reduced sensitivity for low contrast letters[13] and prolonged implicit times of the visually evoked potentials in eyes with NMOSD.[19] We determined the retinal sensitivity of the central 37 points or 10° by macular integrity assessment (MAIA: Topcon Corp, Tokyo, Japan). The MAIA microperimeter is a relatively new instrument equipped with a scanning laser ophthalmoscope with an eye tracking system.[23] MAIA has been used to determine the central retinal sensitivities of eyes with glaucoma,[24] age-related macular degeneration (AMD).[25] macular hole,[26] and other ophthalmic diseases. We selected to use MAIA because standard automated perimetry (SAP) cannot detect the visual dysfunction in eyes with peripapillary RNFL thinning[27], and MAIA microperimetry has been proven to be more sensitive than SAP.[28] The MAIA microperimeter measures the retinal sensitivity for a 37 sector grid covering 10° of the central retina, a 24 sector stimulus grid covering 10° to 2° around the central retina, and a 13 sector stimulus grid covering 2° of the central retina (Fig 2). The fixation target was a red circle of 1° diameter, the stimulus size was Goldmann III, the dynamic range of the stimulus was 36 dB, the background luminance was 4 apostilb (asb), and the maximum luminance was 1000 asb.

### Statistical analyses

All statistical analyses were performed using the R statistical software platform (version 3.2.4)[29]. General estimating equation models were used to determine if the differences of the data between the groups were statistically significant. Mann-Whitney U-tests were also used to determine if the differences in the ages between the groups were statistically significant. Multiple linear regression analysis was carried out to identify the intraretinal layers contributing to average macular thickness. Pearson’s correlation was used to determine the correlation between retinal sensitivity and ONL thickness. Pearson’s correlation was also used to determine the correlation between refractive error and retinal sensitivities. Statistical significance was defined as *P* <0.05

## Results

The mean age of the patients with NMOSD was 49.5 ± 8.9 years and that of the controls was 53.2 ± 5.0 years (*P* = 0.07). The mean refractive error was -1.4 ± 2.1 diopters in the eyes with NMOSD and -0.6 ± 1.4 diopters in the controls (*P =* 0.27). One eye from one patient was excluded from the study due to a thinning of the pRNFL.

**Fig 3:**
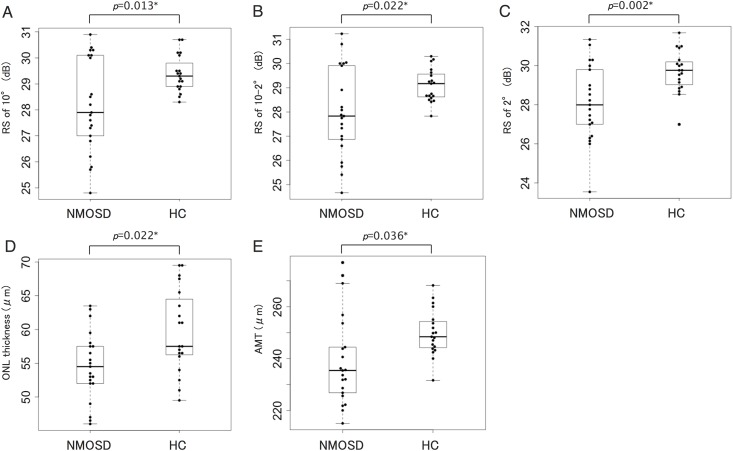
Boxplot and beeswarm plot of retinal sensitivity and intra-retinal layer thickness evaluated by optical coherence tomography. The boxplot and the overlaid beeswarm plot showing the results of retinal sensitivity (RS) and optical coherence tomography (OCT) evaluation obtained from neuromyelitis optica spectrum disorder (NMO) group and healthy control (HC) group. (A) The result of retinal sensitivity (RS) of the central 10° area of retina. (B) The result of RS of the area between central 10° and 2° of the retina. (C) The result of RS evaluated from central 2°. (D), (E) The result of outer nuclear layer (ONL) thickness and average macular thickenss (AMT), respectively. *P <0.05.

The thicknesses of the retinal layers evaluated by OCT and the visual functions of the patients with NMOSD and controls are shown in Table 2. The mean GCIP thickness was 55.7 ± 5.1 μm in the eyes with NMOSD and 57.4 ± 4.3 μm in the eyes of the controls (*P* = 0.28). The mean INL+OPL thickness was 63.7 ± 5.5 μm in the eyes with NMOSD and 63.3 ± 3.0 μm in the eyes of the controls (*P* = 0.78). The mean ONL thickness was 54.6 ± 5.1 μm in the eyes with NMOSD which was significantly thinner than that in the eyes of the controls at 59.8 ± 6.2 μm (*P* = 0.022). The mean AMT was 239.1 ± 17.5 μm in the eyes with NMOSD and 249.9 ± 8.9 μm in the controls. The AMT was significantly thinner in the NMOSD group than in control group (*P* = 0.036) (Fig 3).

**Table 2:** Result of optical coherence tomography and visual function tests.

	No of patients	Laterality	LogMAR BCVA	RS of 10° (dB)	RS of 10–2° (dB)	RS of 2° (dB)	GCIP (μm)	INL+ OPL (μm)	ONL (μm)	AMT (μm)
NMOSD	1	R	0.079	27	26.87	27.08	55.5	57.5	52.5	228.6
		L	0	24.8	25.41	23.54	51.5	56.5	53	226.8
	2	R	0.079	27.3	27	27.77	54.5	65	46.5	222.2
		L	0.079	26.8	26.6	27	55	64	49	221.8
	3	R	0.079	28.5	28.2	29	55.5	54	52	225.6
		L	-0.046	27.8	27.88	30	56	58	53.5	233.6
	4	R	0	25.8	25.75	26.15	56	66.5	63	256.8
		L	0	25.7	24.66	26.3	54.5	69.5	59.5	253.6
	5	R	0.079	28.6	28.91	28	61.5	67.5	55	244.4
		L	0.079	30.1	30	30.3	59.5	66	52	243.8
	6	R	0.079	27.9	27.75	28.23	58	65.5	57.5	235.6
		L	0.079	27.4	27.33	27.46	61.5	63.5	58	236.2
	7	L	0.079	28.2	28.08	28.8	54	64	47	240.6
	8	L	0	26.2	25.91	26.4	39.5	61	46	215
	9	R	0.079	30.4	29.91	28.53	51.5	57.5	54.5	232
		L	0.079	30.3	30.04	31.35	48	57.5	56.5	231.8
	10	L	0.079	27.6	27.83	27.23	62	63	57.5	235.4
	11	R	0.079	30	30	26	57.5	76.5	62	277
		L	0.079	30.1	27.5	30.3	59.5	71.5	63.5	272
	12	R	0.079	30.9	30.8	31.07	58.5	66	55.5	269
		L	0.079	30.3	31.23	29.8	59	67.5	53	220
HC	1	R	0.18	30.7	30.1	30	62	68	68	263.4
		L	0.3	30.2	30.3	31	60	67	57.5	260
	2	R	0	30.1	29.5	31	52	63.5	56	243.2
		L	0.079	29.2	28.75	27	52.5	62	61	245.4
	3	R	0.079	29.4	29.29	29.54	63.5	65	56.5	250
		L	0.079	29.3	29.63	28.69	52.5	62	61	253.6
	4	L	0.079	28.6	28.58	28.54	51	60	51	231.6
	5	R	0.079	29.1	27.83	29.15	58.5	68.5	52.5	240
		L	0.079	28.3	28.67	29.77	62	68	54	242.4
	6	R	0.18	29.1	28.46	30.3	66	63	67.5	268.2
		L	0.18	29.5	29.2	30.1	54	60.5	69.5	261.4
	7	R	0	28.9	28.5	29.61	58.5	64	56.5	251.8
		L	0.079	30.2	29.75	30.92	57.5	61.2	49.5	247
	8	R	0.079	30.7	30.17	31.69	57.5	59	63.5	243.8
		L	0.079	29.5	29.25	30.07	57	59.5	62	244.6
	9	R	0.079	28.8	28.67	28.92	53.5	62	65.5	248.4
		L	0.079	28.9	28.66	29.31	54.5	60.5	69.5	255
	10	R	0	29.4	29.17	29.77	61	64	57.5	248
		L	0.079	28.5	28.42	28.85	56.5	64.5	57	249.8
Average and SD of NMOSD			0.058 ± 0.04	28.18 ± 1.8	27.98 ± 1.8	28.11 ± 1.9	55.64 ± 5.1	63.71 ±5.5	54.62 ± 5.1	239.13 ± 17.5
Average and SD of HC			0.094 ± 0.07	29.39 ± 0.7	29.1 ± 0.7	29.7 ± 1.1	57.37 ± 4.3	63.27 ± 3.0	59.76 ± 6.2	249.87 ± 8.9
*P* value			0.1	0.013*	0.022*	0.002*	0.28	0.78	0.022*	0.036*

AMT = average macular thickness; BCVA = best corrected visual acuity; GCIP = ganglion cell and inner plexiform layer; HC = healthy controls; INL+OPL = inner nuclear layer plus outer plexiform layer; logMAR = logarithm of the minimal angle of resolution; NMOSD = neuromyelitis optica spectrum disorder; OCT = optical coherence tomography; ONL = outer nuclear layer; RS = retinal sensitivity; SD = standard deviation;

*P <0.05.

We performed multiple regression analyses to identify the most contributing intra-retinal layer thickness to the AMT, and there was no significant difference in the contributions among layers. (see S1 Table).

The mean BCVA was 0.06 ± 0.04 logMAR units in the eyes with NMOSD and 0.09 ± 0.07 logMAR units in the controls (*P* = 0.19). The average retinal sensitivity of the central 10° in the eyes with NMOSD was 28.2 ± 1.8 dB which was significantly lower than that in the control group at 29.4 ± 0.7 dB (*P* = 0.013). The average retinal sensitivity in the 10° to 2° area was 28.0 ± 1.8 dB in the NMOSD group which was significantly lower than the 29.1 ± 0.7 dB in the control group (*P* = 0.022). The average retinal sensitivity in the central 2° was 28.1 ± 1.9 dB in the NMOSD group which was significantly lower than the 29.7 ± 1.1 dB in the control group (*P* = 0.002). The correlation between retinal sensitivity and ONL thickness was analyzed, but no significant correlation was observed. (see S2 Table) The correlation between refractive error and retinal sensitivity was also not significant. (see S3 Table)

## Discussion

Our results showed that the ONL and AMT were significantly thinner in eyes with NMOSD than in the control eyes. On the other hand, the thicknesses of the GCIP and INL+OPL in eyes with NMOSD did not differ significantly from that of the control eyes. In addition, our results showed that the retinal sensitivity of the central 10°, 10° to 2°, and 2° were significantly lower in eyes with NMOSD than in the control eyes.

Our findings showed that the ONL was thinner in eyes with NMOSD without prior ON which is evidence that the morphological and sensitivity changes were due to a primary retinal pathology independent of retrograde degeneration.

A thinning of the ONL has been reported in patients with MS and NMOSD. For MS, Saidha et al reported a thinning of the inner nuclear layer (INL) and ONL in eyes without peripapillary RNFL thinning.[21] They suggested that the morphological changes were due to a primary retinal pathology.[21] For NMOSD, Syc et al[17] suggested that the ONL thinning resulted from an inflammation of the Müller cells secondary to a direct attack on Müller cells by anti-AQP4 antibody. On the other hand, Sotirchos et al [13] suggested that the ONL thinning in NMOSD resulted from subclinical optic neuropathy. The thinning of the peripapillary RNFL and GCIP in NMOSD can possibly be caused by an involvement of the anterior visual pathway. Therefore, the cause of the ONL thinning cannot be definitively determined by the findings of these earlier studies.

The difference of the present study from previous NMOSD studies is that we eliminated the effects of retrograde degeneration by excluding patients with reduced peripapillary RNFL thickness. Monteiro et al reported a thinning of the peripapillary RNFL in eyes with NMOSD without prior ON, and it was assumed to be the result of subclinical axonal loss suggesting an involvement of the anterior visual pathway. Because the confounding effect of retrograde degeneration was excluded in the present study, our results indicate that the ONL thinning was most likely independent of any retrograde degeneration.

To identify the factors contributing to the thinning of the AMT, we performed multiple regression analyses of all of the intraretinal layers. The analyses were based on the data of either the right or left eyes to avoid intra-subject and inter-eye dependencies. However, the results of the analyses showed no significant contribution of any retinal layer. (see S1 Table) We believe that the low sample size might have prevented the results from attaining statistical significance.

The microperimetric results showed a reduction of central retinal sensitivities. These findings support the results of an earlier study that reported a reduction of the visual acuity for low contrast letters in eyes with NMOSD without prior optic neuritis.[13] A prolongation of the implicit times of the visual evoked potential in eyes with NMOSD without a prior optic neuritis has been reported,[19] and this would suggest an involvement of the anterior visual pathway in patients with no history of optic neuritis. Because retrograde degeneration was excluded from our study, we believe that the reduced retinal sensitivity was most likely not due to retrograde degeneration. The results also indicate that microperimetry is useful for detecting subclinical impairments of visual function because conventional visual acuity measurements may not detect a reduction of the BCVA.

In the seronegative NMOSD patients of the present study, the antibody might not be detected leaving the possibility of antibody existing in these patients. Jiao et al[30] reported that the sensitivity of serological status of NMOSD varies between the assays, and the most sensitive assay was reported to be fluorescence-activated cell sorting assay which we did not conduct. Zajda et al[31] have also reported that seronegative NMOSD patients had lower levels of serum aquaporin 4 concentration than multiple sclerosis and control group, suggesting that serum anti-aquaporin 4 can be used as a diagnostic marker of NMOSD. These new techniques should be used in future studies to ensure an accurate serological status of the subjects.

Previous study have reported that anti-myelin oligodendrocyte glycoprotein (MOG) antibody is detected in subgroup of seronegative NMOSD[32]. The course of the disease is reported to be favorable in NMOSD patients with positive MOG antibody[33], but whether the subgroup can be categorized as a distinct disease from NMOSD remains to be determined[34]. In the preset study, seronegative NMOSD patients might be related to anti-MOG antibody, but we did not test for anti-MOG antibody. Future studies should include the test for this increasingly recognized antibody.

The results of earlier studies showed that low to moderate myopia can affect the results of retinal sensitivity measurements. Gella et al [35] have reported that the retinal sensitivity is positively correlated with the refractive error. In the present study, the difference in the refractive errors between NMOSD and HC group was not significant. This then allowed a comparison of the data between the two groups. In addition, the correlations between the refractive error and the retinal sensitivity were also not significant (see S3 Table). Thus, we suggest that the effect of the refractive error was minimal in our patients.

The thickness of the ONL has been reported to be significantly correlated with different visual functions,[36] but we did not confirm the correlation between retinal sensitivities and ONL thickness. (see S2 Table) This result may be due to low sample size, and the larger sample is needed in future studies to reconfirm the function-morphological correlation.

The exact mechanism causing the primary retinal pathology was not determined, but previous reports suggested that the retinal pathology of NMOSD was related to the Müller cells. [11, 21] AQP4 is a water channel mainly expressed on the foot processes of astrocytes in the central nervous system,[3, 37] Pathological studies have shown that astrocytes were preferentially targeted in eyes with NMOSD.[38] In the retina, Müller cells are the major astrocytes, and it has been shown that AQP4 is expressed on human Müller cells.[39] These findings led to the hypothesis that retinal Müller cells are directly attacked by anti-AQP4 antibody.

Müller cells are specialized glial cells which span the entire thickness of the retina.[40] The main functions of Müller cells are maintaining the ion and water homeostasis of the retina and providing metabolic support and nutrition to the retinal neurons.[40]

Once Müller cells are attacked by anti-AQP4 antibodies, it is possible that retinal neurons would undergo secondary damage which would then result in the loss of neurons. Any neurons in retina could suffer from Müller cell dysfunction because Müller cells span the entire thickness of retina. However, we excluded eyes with inner retinal layer thinning to focus on changes of the outer retinal layers including the ONL. To evaluate the effect of primary retinal pathology on the inner retinal layers, pathological studies on animal models of NMOSD are essential.

For the anti-AQP4 antibody to attack Müller cells, the antibody must have access to the Müller cells. Although vascular abnormalities including retinal artery attenuation have been reported in eyes with NMO,[41] leakage from the retinal vessels has not been demonstrated.[41] The blood-brain barrier has been reported to be compromised in eyes with NMOSD,[42] and we suggest that the blood-retinal barrier might also be compromised giving access of anti-AQP4 antibody to the retinal tissues.

**Fig 4:**
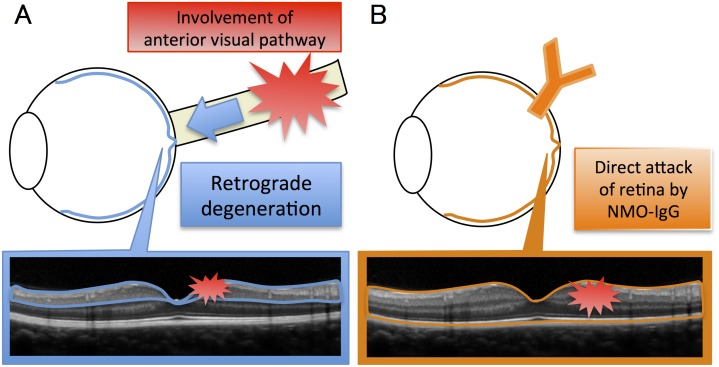
Hypothesis of different mechanisms between retrograde degeneration and direct retinal pathology. (A) In retrograde degeneration of neuromyelitis optica spectrum disorder (NMOSD), involvement of anterior visual pathway results in a thinning of the inner retinal layers including the retinal nerve fiber layer(RNFL) and ganglion cell-inner plexiform layer (GCIP). (B) In direct retinal pathology of NMOSD, a direct attack of Müller cells by anti-aquaporin 4 (AQP4) antibody results in a secondary loss of retinal neurons including ONL thinning.

Thus, our results suggest that the alterations of retinas in eyes with NMOSD are due to a primary retinal pathology. In a previous study, the thinning of the inner retinal layer in eyes with MS and NMOSD was explained by retrograde degeneration due to optic neuritis or subclinical optic neuritis.[13] The results of our study suggest a pathology independent of retrograde degeneration, and the results suggest a primary retinal pathology including a direct attack of the retina by anti-AQP4 antibody (Fig 4).

Our study has several limitations. First, the number of NMOSD patients was small. The small population size limited the strength of the statistical analysis and the likelihood of reaching the statistical significant threshold. A study with a larger cohort is needed to confirm the results of this study.

Second, lack of angiographic data prevented a confirmation of leakage from the retinal vessels and the access of anti-AQP4 antibody to retinal tissue. Future studies are needed to evaluate the integrity of the blood-retinal barrier and the retinal vascular system.

Third, there was a lack of pathological examinations of eyes with NMOSD. Future studies are needed to include pathological examinations to confirm the existence of anti-AQP4 antibody in the retinal tissues and morphological changes of the retina.

Fourth, the results have a limitation due to the evaluation method. Oberwahrenbrock et al and Otani et al reported that the ONL thickness might be underestimated by the high reflection of Henle’s fiber in outer plexiform layer. [43, 44] Lujan et al also reported that the ONL thickness measurements were affected by the beam angle of the OCT due to the existence of Henle fiber layer, and also that directional OCT is needed for accurate measurements. [45] Because our method was not designed to control the incidence of the OCT beam, caution is needed in interpreting our data on the ONL.

Finally, we were not able to conclude that anti-AQP4 antibodies were directly attacking the retina in eyes with NMOSD, and histopathological examinations would enable confirming our hypothesis.

In conclusion, our results showed that the retinal sensitivity was significantly reduced along with a significant thinning of the ONL in eyes with NMOSD without prior optic neuritis. We suggest that the cause of ONL thinning is a primary retinal pathology, and the decreased retinal sensitivity might be secondary to the ONL thinning.

## Supporting Information

S1 Table Results of multiple regression analyses of different retinal layers contributing to the average macular thickness. (DOCX)

S2 Table Correlations between retinal sensitivities and outer nuclear layer thickness. (DOCX)

S3 Table Correlation between refractive error and retinal sensitivities. (DOCX)

## Abbreviations

AMT: average macular thickness
NMOSD: neuromyelitis optica spectrum disorder
ON: optic neuritis
ONL: outer retinal layer
RNFL: retinal nerve fiber layer
SD-OCT: spectral-domain optical coherence tomography
