# Supplementary material for: Retinal Morphology and Sensitivity Are Primarily Impaired in Eyes with Neuromyelitis Optica Spectrum Disorder (NMOSD)
Source: PLoS One. 2016 Dec 9;11(12):e0167473. doi: 10.1371/journal.pone.0167473 (PMC5147908; doi:10.1371/journal.pone.0167473)
Supplement: S1 Table — (DOCX) [file pone.0167473.s001.docx]

**S1 Table**. Results of multiple regression analyses of different intraretinal layers contributing to the average macular thickness.

|  | Intraretinal  layers | Coefficients | *P* |
| --- | --- | --- | --- |
| Data from  Right eyes*  (n=9) | GCIP | 0.49 | 0.80 |
|  | INL+OPL | 1.35 | 0.19 |
|  | ONL | 1.89 | 0.13 |
| Data from Left eyes**  (n=12) | GCIP | -0.10 | 0.88 |
|  | INL+OPL | 1.35 | 0.13 |
|  | ONL | 1.74 | 0.051 |

*R^2^ = 0.75 **R^2^ = 0.65

Abbreviations: GCIP = ganglion cell and inner plexiform layer, INL+OPL = inner nuclear layer and outer plexiform layer, ONL = outer nuclear layer.

The analysis was based on the data of either the right eyes or left eyes to avoid intra-subject inter-eye dependencies.
