# Supplementary material for: Retinal Morphology and Sensitivity Are Primarily Impaired in Eyes with Neuromyelitis Optica Spectrum Disorder (NMOSD)
Source: PLoS One. 2016 Dec 9;11(12):e0167473. doi: 10.1371/journal.pone.0167473 (PMC5147908; doi:10.1371/journal.pone.0167473)
Supplement: S2 Table — (DOCX) [file pone.0167473.s002.docx]

**S2 Table**. Correlations between retinal sensitivities and outer nuclear layer thickness.

|  |  | r | *P* |
| --- | --- | --- | --- |
| Data from  Right eyes  (n=9) | RS of 10° vs ONL | 0.024 | 0.95 |
|  | RS of 10°-2° vs ONL | 0.078 | 0.84 |
|  | RS of 2° vs ONL | -0.41 | 0.27 |
| Data fom  Left eyes  (n=12) | RS of 10° vs ONL | 0.23 | 0.47 |
|  | RS of 10°-2° vs ONL | -0.023 | 0.94 |
|  | RS of 2° vs ONL | 0.19 | 0.55 |

Abbreviations: RS = retinal sensitivity, ONL = outer nuclear layer.

Pearson’s correlation was used to determine the correlations.

The analysis was based on the data of either the right eyes or left eyes to avoid intra-subject inter-eye dependencies.
