# Supplementary material for: Retinal Morphology and Sensitivity Are Primarily Impaired in Eyes with Neuromyelitis Optica Spectrum Disorder (NMOSD)
Source: PLoS One. 2016 Dec 9;11(12):e0167473. doi: 10.1371/journal.pone.0167473 (PMC5147908; doi:10.1371/journal.pone.0167473)
Supplement: S3 Table — (DOCX) [file pone.0167473.s003.docx]

**S3 Table**. Correlation between refractive error and retinal sensitivities

|  |  | r | *P* |
| --- | --- | --- | --- |
| Data from right eyes (n=9) | RE vs RS of 10° | 0.041 | 0.92 |
|  | RE vs RS of 10°-2° | 0.023 | 0.95 |
|  | RE vs RS of 2° | -0.58 | 0.88 |
| Data from left eyes (n=12) | RE vs RS of 10° | 0.022 | 0.95 |
|  | RE vs RS of 10°-2° | -0.096 | 0.77 |
|  | RE vs RS of 2° | -0.0012 | 0.99 |

Abbreviations: RE = refractive error, RS = retinal sensitivity.

Pearson’s correlation was used to evaluate the correlation.

The analysis was based on the data of either the right eyes or left eyes to avoid intra-subject inter-eye dependencies.
